# Qualitative Perfusion Cardiac Magnetic Resonance Imaging Lacks Sensitivity in Detecting Cardiac Allograft Vasculopathy

**DOI:** 10.4021/cr105w

**Published:** 2011-11-20

**Authors:** Monica Colvin-Adams, Salam Petros, Ganesh Raveendran, Emil Missov, Eduardo Medina, Robert Wilson

**Affiliations:** a University of Minnesota, Cardiovascular Division, Minneapolis, MN 55455, USA

**Keywords:** MRI, Cardiac allograft vasculopathy, Cardiac transplantation

## Abstract

**Background:**

Cardiac allograft vasculopathy (CAV) is a major complication after heart transplantation, requiring frequent surveillance angiography. Though cardiac angiography is the gold standard, it is insensitive in detecting transplant vasculopathy and invasive. Perfusion MRI provides a noninvasive alternative and possibly a useful modality for studying CAV. We sought to compare the accuracy of qualitative perfusion MRI to coronary angiography in detecting CAV.

**Methods:**

A retrospective analysis was performed in 68 heart transplant recipients who had simultaneous surveillance cardiac MRI and coronary angiogram and who underwent transplantation between 2000 and 2007. We compared results of qualitative MRI to those of the cardiac angiogram. Sensitivity and specificity of MR were calculated.

**Results:**

Sixty-eight patients underwent both cardiac MRI and coronary angiogram. 73.5% were male; mean age was 45.37 ± 14 years. Mean duration of heart transplantation was 7.9 ± 5.2 years. The mean ejection fraction was 55% in the patients without CAV and 57.4% in those with CAV. There were 48 normal and 24 abnormal MRI studies. The overall sensitivity was 41% and specificity was 74%.

**Conclusions:**

Qualitative assessment of perfusion cardiac MR has low sensitivity and moderate specificity for detecting CAV. The sensitivity of MRI was slightly improved with severity of disease.

## Background

In the United States, heart failure is an epidemic affecting over 5 000 000 people. It is estimated that between 100 000 and 200 000 people suffer from end-stage heart failure [1]. Cardiac transplantation has emerged as the definitive therapy for patients with end-stage heart failure, however only 2000 - 2300 transplants/year have been performed nationally over the last 10 years (Based on OPTN data as of November 13, 2009). Success rates have improved since the inception of transplant, and the median survival has increased to 10 - 13 years. (Based on ISHLT Registry data, 2009) Cardiac allograft vasculopathy (CAV), one of the leading causes of death after transplant, is a major limitation to long-term survival in cardiac transplant recipients and affects between 30 - 60% of recipients by year 5 [2-5]. Most centers perform routine surveillance angiograms, however, by the time CAV is visible on angiogram, it is typically advanced. Thus, there is a need for early detection and, ideally noninvasive, methods.

In comparison to native coronary artery disease, which tends to be focal, CAV is a diffuse process that involves intimal thickening of all of the coronary vessels. As a result, angiography is less sensitive in detecting CAV due to its ability to only measure luminal size and area and its poor discrimination of small arteries [6]. To make an accurate assessment, comparisons of serial angiograms must be performed to detect diminution of vessels. Intravascular ultrasound (IVUS) has the ability to assess both wall and lumen of the proximal large arteries, however the cost, lack of expertise, and limited ability to image secondary and tertiary vessels hinders its widespread implementation. Despite the challenges associated with angiography, it remains the standard for detection of coronary disease in transplant recipients. Magnetic resonance perfusion imaging using gadolinium-based contrast agents has been validated as a versatile noninvasive clinical tool to quantify myocardial perfusion [7, 8]. We sought to evaluate the sensitivity and specificity of qualitative cardiac MRI in the evaluation of cardiac allograft vasculopathy.

## Methods

### Subjects

We performed a retrospective analysis of 72 paired coronary angiograms and perfusion MRIs in 68 heart transplant recipients to determine the sensitivity and specificity of MRI in detecting CAV on angiogram. All heart transplant recipients at our program undergo yearly evaluation for CAV with coronary angiogram during the first 4 years after transplant. After the first 4 years, coronary angiogram is performed every other year alternating with a noninvasive study, either exercise echocardiogram or a thallium study, for the life of the transplant recipient. Between November 2000 and June 2007, patients also underwent simultaneous (within one month) perfusion MRI as part of the heart transplant protocol. Four patients had two sets of MRI-angiogram studies; a total of 72 MRI-angiogram pairs were included in the analysis. We compared the qualitative findings on MRI to the results of the associated angiogram. Each angiogram was evaluated by 2 independent interventional cardiologists. MRIs were read by a single MRI specialist blinded to the results of the angiograms. We compared sensitivity and specificity of MRI in detecting the angiographic evidence of cardiac allograft vasculopathy. This analysis was approved by the Institutional Review Board at the University of Minnesota.

### Magnetic resonance image acquisition

A 1.5-T magnetic resonance scanner (Siemens Vision, Erlangen,Germany) and a phase-array body coil were used for imaging. Scout images determined the short- and long-axis views of the heart. Perfusion imaging during rest and adenosine-induced hyperemia was then performed. Adenosine was titrated in three steps to a maximum dose of 140 mg/kg/minute for three minutes. Image acquisition was started 1 minute after the start of the of the maximal infusion rate.

Perfusion was determined in three LV short-axis slices using a standard protocol. A single shot gradient-echo sequence with saturation-recovery magnetization preparation for T1 weighting and linear k-spacing was used for imaging. The parameters were set to repetition time/echo time/flip angle of 2.4 ms/1.2 ms/18° and a slice thickness of 10 mm.

Temporal resolution allowed acquisition of one image in each of the three selected slices within one heart beat up to a heart rate of 110 beats/minute. Sixty images per slice location were acquired with a spatial resolution of 2 to 3 mm. Three heart beats after initiation of the sequence, a compact bolus of 0.03 mmoL/kg bodyweight gadolinium-DTPA (Magnevist, Berlex, New Jersey) was injected over an antecubital vein at a rate of 7 mL/s using a power injector (MedRad, Pennsylvania). Heart rate, peripheral blood pressure, and oxygen saturation were continuously monitored during the magnetic resonance procedure. Images were analyzed by a single reader who was blinded to the patient’s name, clinical status, and results from the invasive measurements. Perfusion studies were analyzed (ARGUS Software, Siemens, Iselin, New Jersey) via qualitative analysis. Both rest and stress images were acquired. Areas of hypoperfusion were assigned to coronary territories using the American Heart Association coronary arterial segment model.

### Coronary angiogram

Selective angiography was performed yearly during the first 4 years and every other year as part of the post-transplantation protocol. Coronary angiogram was performed using standard angiography techniques and projections. All angiograms were reviewed in a blinded fashion by 2 interventional cardiologists. Though coronary arteries were assessed for both diffuse and focal lesions, cardiac allograft vasculopathy was defined as a 25% or greater stenosis in a major epicardial artery.

### Statistical analysis

Sensitivity and specificity of MRI in detecting cardiac allograft vasculopathy was calculated. In addition, sensitivity and specificity based on severity of CAV was determined.

## Results

### Demographics

Eventy-four percent of patients were male. The mean age was 46 years (range 13 - 65). No patients who had undergone multi-organ transplant were included. One patient had a second heart transplant at the time the studies were obtained. The mean duration between heart transplant and angiogram/MRI was 8 years (range 1 - 23). The mean ejection fraction was 56%. Thirty-four percent had cardiac allograft vasculopathy by the aforementioned definition. (Table 1)

**Table 1:** The Demographic Characteristics of Patients

Demographic (n = 68)	Number
Gender (% male)	74
Age (years)	45 ± 14
Time since HTx (years)	7.9 ± 5.2
Retransplants	1
Mean EF (%)	55.6 ± 8.6
Angiographic CAV present (%)	47
CAV grade (%)	
0	19.4
1	33.3
2	32
3	4.2
4	11.1

Twenty-four MRIs were classified as abnormal; 10 of the MRIs were false positives. There were 48 normal cardiac perfusion MRI studies; twenty patients with normal MRIs had mild CAV (grade 1 - 3) and four subjects had grade 4 CAV.

**Figure 1:**
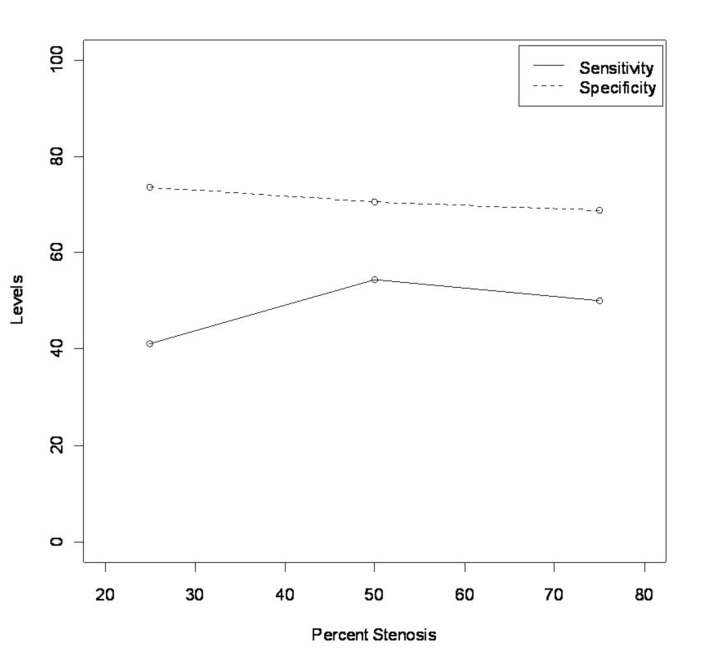
Change in specificity and sensitivity with degree of stenosis.

We evaluated the sensitivity and specificity of MRI in detecting cardiac allograft vasculopathy, defined as 25% or greater lesion. The overall sensitivity was 41% and the specificity was 74%. We further evaluated the sensitivity and specificity by severity of CAV. There was a non-significant biphasic increase in sensitivity and a decrease in specificity with increased severity of CAV. (Fig. 1)

### False negatives

There were 20 studies with normal MRIs and CAV present on angiogram (28% false negative rate). Of these, 4 had grade four disease. One patient did not receive adenosine and one had no response to adenosine. We compared the angiographic location of disease to patients with grade 4 CAV and abnormal MRIs. Though the numbers are small for comparison, there was a tendency toward significant proximal vasculopathy in those patients with a true positive MRI. Those who had false negative MRIs tended to have diffuse disease on angiogram. Collateralization did not explain the false negative MRIs since both groups had evidence of collateralization.

### False positives

Ten patients were found to have a false positive MRI. The rate of subendocardial ischemia in false positive studies was 60%. We attribute this to the possibility of microcirculatory disease, however are unable to substantiate this without intravascular ultrasound and provocative testing.

## Discussion

In our study, perfusion MRI detected cardiac allograft vasculopathy, defined as a stenosis of 25% or greater in a major epicardial artery, with a sensitivity of 41% and specificity of 74%. When severity of CAV was assessed, sensitivity increased slightly and specificity decreased.

Current noninvasive methods for evaluating cardiac allograft vasculopathy include echocardiography and radionuclide imaging techniques. Dobutamine stress echocardiogram is perhaps the most widely used noninvasive test for evaluation of CAV. In general, sensitivity and specificity of DSE in heart transplant recipients has been acceptable with sensitivity being reported between 79 - 95% and specificity from 55% to 91% when compared to angiography [9, 10]. Furthermore, DSE appears to have prognostic value. Akosah et al. demonstrated that an abnormal DSE was associated with a 33% chance of a cardiac event in heart transplant recipients, defined as myocardial infarction, angina or heart failure. Serial DSE also predicts outcome, with worsening on serial DSE being associated with more than a 7-fold increase in risk of events [11, 12].

While highly specific (77 - 100%), stress ECG has extremely poor sensitivity (0 - 38%). Similarly, thallium scintigraphy is highly specific with moderate to poor sensitivity. Multi-detector computed tomography (MDCT) with adaptive multi-segment reconstruction has been shown to detect CAV with 86% sensitivity and 99% specificity [13]. Major challenges to MDCT include achieving a desirable heart rate in heart transplant patients, contrast allergy, and the significant amount of radiation required.

There are several limitations to noninvasive testing in heart transplant recipients. Attaining adequate heart rate for exercise studies may be difficult in heart transplant recipients due to medications and limited exercise capacity. Furthermore, the gold standard used for comparison, that is, coronary angiogram, is in itself, insensitive in identifying cardiac allograft vasculopathy. Studies that rely on differences in perfusion may be insensitive in detecting diffuse cardiac allograft vasculopathy.

Cardiac perfusion MRI, in contrast to exercise testing, does not rely on achievement of a target heart rate. It provides superior spatial resolution when compared to radioisotope techniques and does not require the use of radiation. Perfusion MRI is greatly enhanced with the use of quantitative techniques. In patients with suspected native coronary artery disease, Al-Saadi et al demonstrated a sensitivity of 90%, specificity of 83% and a diagnostic accuracy of 87% for the detection of native CAD using a myocardial perfusion reserve cutoff of 1.5 [14]. Schwitter and colleagues detected a sensitivity of 87% and sensitivity of 85% when compared to angiographic native CAD (defined as a lesion greater than 50%) [15].

Diagnosis of cardiac allograft vasculopathy using noninvasive techniques, however, represents a challenge due to the inherent differences between native CAD and CAV and its expression in the vasculature. Significant CAV may be present even in the presence of a non-significant stenosis in the epicardial arteries. Wilson et al demonstrated that discrete coronary lesions producing less than 70% area stenosis and less than 50% diameter stenosis do not produce hemodynamically significant reductions in coronary flow reserve (CFR). Furthermore, there is poor correlation between degree of stenosis and CFR in patients with diffuse coronary artery disease [16]. Heart transplant recipients, however, may have lesser degrees of stenosis on angiogram which may be prognostically significant yet not produce decreases in CFR. It has been demonstrated that in heart transplant recipients, diffuse concentric narrowing (> 50%) in all 3 main coronary arteries and a CFR < 2.5 will produce a significant reduction in myocardial perfusion reserve and endo-/epicardial perfusion ratio (P < 0.01 for both) on MRI, however it is not clear whether lesser degrees of stenosis will produce changes on quantitative or qualitative perfusion MRI [17, 18].

Our analysis compared qualitative perfusion MRI to coronary angiogram. We did not incorporate quantitative techniques as these are not widely used and thus would not reflect practice in most clinical settings. In our series, we detected a low sensitivity and moderate specificity that was not comparable to dobutamine stress echocardiogram, which has been reported as having excellent sensitivity and specificity in CAV. In addition, sensitivity appeared to improve with severity of CAV but declined with the most severe disease. One explanation for this decline in sensitivity is the decreased ability to detect relative differences in perfusion in advanced and diffuse coronary disease. Specificity also declined, though not significantly, with severity of disease.

Based on the low sensitivity of cardiac perfusion MRI in detecting CAV, we conclude that qualitative MRI assessment alone cannot be recommended as a screening tool for cardiac allograft vasculopathy.

### Limitations

There are several limitations to this study that should be noted. In this study we evaluated qualitative MRI without the use of quantitative techniques, however quantitative analyses may have provided a better assessment of subendocardial ischemia and microvascular disease which may precede the development of epicardial disease. Nevertheless, qualitative MRI is widely utilized, thus we believe this approach was an accurate evaluation of actual clinical practice. We acknowledge the limitations of using angiogram as the gold standard. CAV involving subendocardial vessels or diffuse, non-stenotic disease is not easily detected on angiogram. It is possible, however, that these lesions may cause changes in perfusion thus resulting in a “false-positive” result on the MRI. Intravascular ultrasound and functional testing may have improved the invasive detection of CAV and small vessel disease. This may have allowed a better determination of the cause of the false positive MRI results. On the other hand, the false negative rate was 28%, and appeared to be associated with the presence of diffuse disease on angiogram. Thus, having a diagnosis by IVUS may not have necessarily improved the specificity. We did not evaluate sensitivity and specificity by type and location of lesion, which may have provided different results.

### Conclusions

Qualitative perfusion MRI is insensitive in the detection of cardiac allograft vasculopathy and should not be used as a screening study in the evaluation of heart transplant recipients. Quantitative assessments may enhance the ability of MRI to detect CAV and thus represent a more reliable noninvasive diagnostic tool. Furthermore, in planning future studies to evaluate noninvasive techniques in the diagnosis of CAV, consideration should be given to using intravascular ultrasound as the gold standard.
